# Analysis of Eligibility for Lung Cancer Screening by Race After 2021 Changes to US Preventive Services Task Force Screening Guidelines

**DOI:** 10.1001/jamanetworkopen.2022.29741

**Published:** 2022-09-02

**Authors:** Laura C. Pinheiro, Lauren Groner, Orysya Soroka, Ashley E. Prosper, Kellie Jack, Rulla M. Tamimi, Monika Safford, Erica Phillips

**Affiliations:** 1 Division of General Internal Medicine, Department of Medicine, Weill Cornell Medicine, New York–Presbyterian Hospital, New York; 2 Sandra and Edward Meyer Cancer Center, Weill Cornell Medicine, New York–Presbyterian Hospital, New York; 3 Department of Population Health Sciences, Weill Cornell Medicine, New York, New York; 4 Department of Radiology, Weill Cornell Medicine, New York–Presbyterian Hospital, New York; 5 Department of Radiological Sciences, David Geffen School of Medicine, University of California, Los Angeles

## Abstract

**Importance:**

Lung cancer incidence and mortality have disproportionate consequences for racial and ethnic minority populations. The extent to which the 2021 changes to the US Preventive Services Task Force (USPSTF) screening guidelines have reduced the racial disparity gap in lung cancer screening eligibility is not known.

**Objective:**

To assess the consequences of the changes in USPSTF low-dose computed tomography eligibility criteria for lung cancer screening between 2013 and 2021 among Black and White community-dwelling adults.

**Design, Setting, and Participants:**

This cohort study analyzed data from the Reasons for Geographic and Racial Differences in Stroke study, a prospective longitudinal cohort study of community-dwelling Black and White adults 45 years and older who were initially recruited across the US between January 2003 and October 2007, with ongoing follow-up. All participants who would have been potentially eligible for lung cancer screening based on the 2021 USPSTF guidelines (N = 14 285) were included. Follow-up data for the current cohort study were collected and analyzed between January 2013 and December 2017, with final analysis performed in 2021.

**Exposures:**

Self-reported Black vs White race.

**Primary Outcomes and Measures:**

Differences in the proportion of Black vs White participants eligible for lung cancer screening according to 2013 and 2021 guidelines were assessed using modified Poisson models with robust SEs. Associations between important covariates (demographic characteristics and social factors associated with health), including interaction and dissimilarity indices (2 measures of residential segregation), and differences in screening eligibility were also examined.

**Results:**

Among 14 285 participants (mean [SD] age, 64.7 [7.5] years; 7675 men [53.7%]), 5787 (40.5%) self-identified as Black and 8498 (59.5%) as White. Based on the 2013 USPSTF guidelines, 1109 of 5787 Black participants (19.2%) and 2313 of 8498 White participants (27.2%) were eligible for lung cancer screening (difference, −8.06 percentage points; 95% CI, −9.44 to −6.67 percentage points). Based on the 2021 guidelines, 1667 of 5787 Black participants (28.8%) and 2940 of 8498 White participants (34.6%) were eligible for screening (difference, −5.73 percentage points; 95% CI, −7.28 to −4.19 percentage points). After adjustment for differences in individual characteristics and residential segregation, the 2013 difference in screening eligibility among Black vs White participants was −12.66 percentage points (95% CI, −14.71 to −10.61 percentage points), and the 2021 difference was −12.15 percentage points (95% CI, −14.37 to −9.93 percentage points).

**Conclusions and Relevance:**

In this study, 2021 changes to the USPSTF lung cancer screening guidelines were associated with reductions in but not elimination of existing eligibility disparities in lung cancer screening among Black and White adults. These findings suggest that accounting for factors beyond age and pack-years of smoking is needed when tailoring guidelines to improve screening eligibility among groups at high risk of lung cancer.

## Key Points

**Question:**

What consequences have the 2021 changes to the US Preventive Services Task Force screening guidelines for lung cancer had for the racial gap in lung cancer screening eligibility between Black and White community-dwelling adults?

**Findings:**

In this cohort study of 14 285 Black and White adult participants in the Reasons for Geographic and Racial Differences in Stroke study, after adjustment for individual characteristics and important social factors associated with health (eg, residential segregation), screening guideline changes were associated with a difference in lung cancer screening eligibility among Black and White individuals of −12.7 percentage points in 2013 and −12.2 percentage points in 2021.

**Meaning:**

These findings suggest that although expansion of the lung cancer screening eligibility criteria was important to address racial differences in screening, without reform to policies with the explicit goal of eliminating structural factors such as residential segregation, changes in screening guidelines may only minimally improve existing racial gaps in eligibility.

## Teaser

This cohort study uses data from the Reasons for Geographic and Racial Differences in Stroke study to assess consequences of the 2021 changes to the US Preventive Services Task Force eligibility criteria for lung cancer screening among Black and White adults.

## Introduction

Lung cancer is the leading cause of cancer-related death and the second most diagnosed cancer in the US.^1^ Despite similar smoking prevalence rates among Black (16.7%) and White (16.6%) adults in the US, there are substantial differences between the 2 groups regarding lung cancer incidence and mortality.^2^ Black individuals, particularly men, have higher age-adjusted lung cancer incidence and mortality rates than White individuals and those from other racial groups.^3^ Overall, there have been steady decreases in the incidence of lung cancer over the past 2 decades and mortality rates over the last 3 decades. Decreases have been primarily associated with (1) successful tobacco control and smoking cessation initiatives, (2) early detection via low-dose computed tomography (LDCT) lung cancer screening, and (3) improvements in treatment, particularly of non–small cell lung cancer.^4^ However, these patterns have favored individuals with high socioeconomic status and non-Hispanic White race and ethnicity. Thus, substantial disparities between Black and White individuals remain with regard to the rates of early lung cancer detection.

Underrepresentation of Black adults in the landmark National Lung Screening Trial (NLST),^5^ a large-scale randomized clinical trial of lung cancer screening, has been identified as 1 factor responsible for the racial gap in lung cancer screening eligibility. The NLST showed that screening with LDCT reduced lung cancer–specific mortality by 20% and all-cause mortality by 6% compared with chest radiography.^5^ A secondary analysis of NLST data found that Black participants derived the most substantial mortality benefit from LDCT screening (hazard ratio, 0.61 for Black individuals vs 0.86 for White individuals).^6^ In response to the findings of the NLST clinical trial, the US Preventive Services Task Force (USPSTF) recommended lung cancer screening based on age and duration of smoking in its 2013 guidelines.^7^ The USPSTF recommended that individuals aged 55 to 80 years with at least a 30 pack-year smoking history who either currently smoked or quit smoking within the last 15 years be screened for lung cancer using LDCT. These guidelines were updated in 2021,^8^ reducing the minimum age from 55 years to 50 years and smoking intensity from 30 pack-years to 20 pack-years. The recent changes have been met with enthusiasm in the hopes that they would improve screening rates among individuals, such as Black adults, who are less often eligible for lung cancer screening despite developing lung cancer at younger ages and after fewer pack-years of smoking.^9,10^ Although the long-term consequences of the 2021 guidelines are not yet known, the fixed criteria based on smoking history and age alone have not accounted for additional risks from social factors associated with health.

It has been well established that social factors associated with health, such as lack of health insurance, low educational attainment, and low income, are associated with worse cancer outcomes.^11,12,13^ However, these factors are components of broader socioeconomic and political policies (ie, components of structural racism) that perpetuate inequality. One example is residential segregation, the physical separation of groups based on the social construct of race that was fundamentally designed to prevent social interactions between White and Black individuals.^14^ Residential segregation has been associated with higher rates of multiple chronic conditions, including cancer.^15,16,17,18^ The consequences of residential segregation and other social factors associated with health have not been explicitly examined in the context of recent changes to lung cancer screening recommendations. To address this evidence gap, we used data from the prospective Reasons for Geographic and Racial Differences in Stroke (REGARDS) cohort study with 2 objectives: (1) to describe racial differences in eligibility for LDCT screening based on the 2013 USPSTF guidelines and estimate how many individuals would theoretically become eligible based on the 2021 changes to the USPSTF guidelines and (2) to evaluate the consequences of residential segregation and several other social factors associated with health (ie, annual household income, health insurance status, educational level, and social network size) for disparities in lung cancer screening eligibility based on 2013 vs 2021 guidelines.

## Methods

### Data Sources and Study Population

Full details about the design of the REGARDS study have been published previously.^19^ In brief, the REGARDS study is a prospective longitudinal cohort study of 30 239 community-dwelling adults 45 years and older who were initially recruited across the 48 contiguous US states and the District of Columbia between January 2003 and October 2007, with ongoing follow-up. Individuals were eligible to participate in the REGARDS study if they self-identified as non-Hispanic Black or White race and ethnicity because the most substantial stroke-related racial disparities existed between these 2 groups at the time of study design. Individuals were excluded if they self-reported medical conditions that would prevent long-term participation (eg, cancer) or were currently on a waiting list for nursing home placement. Study methods were reviewed and approved by the institutional review boards of the University of Alabama and Weill Cornell. Follow-up data for the current cohort study were collected between January 2013 and December 2017, with final analysis performed in 2021. All participants provided written informed consent. This study followed the Strengthening the Reporting of Observational Studies in Epidemiology (STROBE) reporting guideline for cohort studies.

The REGARDS study recruited 56% of participants from the stroke belt, an area in the southeastern US with high stroke mortality rates. The buckle of the stroke belt refers to the coastal plains of Georgia, North Carolina, and South Carolina (21% of the total sample); the remainder of the stroke belt consists of other areas in Alabama, Arkansas, Louisiana, Mississippi, North Carolina, and Tennessee (35% of the sample). The remaining 44% of the sample was recruited from the other 40 continental US states. An initial telephone interview was used to ascertain sociodemographic information (age, sex, race and ethnicity, educational attainment, health insurance status, and annual household income), lifestyle information, self-reported health status, and medical history.

### Study Sample

The analytic sample in this study included 29 279 participants in the REGARDS study for whom information about smoking status at baseline, age at initiation of smoking, age at cessation of smoking, and number of pack-years of smoking was available. We subsequently excluded 13 604 individuals who had never smoked and 1390 individuals who were younger than 50 years or older than 80 years at baseline because they would not be theoretically eligible for LDCT screening based on either the 2013 or 2021 USPSTF guidelines. The final sample comprised 14 285 participants. The participant exclusion flowchart is shown in the Figure.

**Figure.**
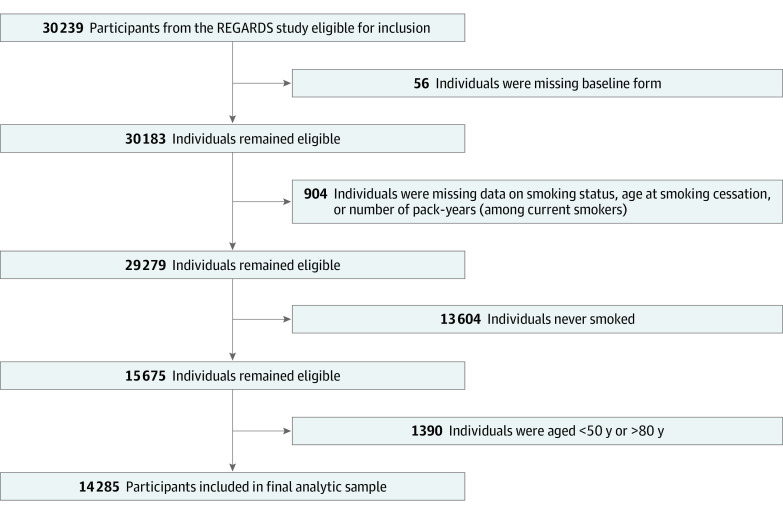
Participant Exclusion Flowchart REGARDS indicates Reasons for Geographic and Racial Differences in Stroke.

### Primary Outcome and Covariates

We examined LDCT screening eligibility based on the 2013 and 2021 USPSTF guidelines.^20^ Self-reported Black vs White race was the primary exposure variable.

Covariate selection was guided by the Commission on Social Determinants of Health framework proposed by the World Health Organization.^21^ This framework combines theoretical models to explain the underlying factors associated with health inequities and identify areas in which to intervene. For this analysis, we focused on 2 categories: (1) socioeconomic and political (residential segregation) and (2) social hierarchy and class (sex, highest level of education, health insurance status, and annual household income).

Additional covariates included age at baseline, marital status, rurality of residence, region of residence, mental well-being, and social network size. Studies have found that smoking has a high comorbidity rate among adults with lower mental well-being (eg, depression or anxiety).^22^ Mental well-being was measured using the mental component summary of the 12-item Short Form Health Survey, a generic health-related quality of life instrument (score range, 0-100, with higher scores indicating better mental well-being).^23^ Larger social networks (ie, number of close friends and relatives) have been associated with favorable health behaviors, such as smoking cessation and reduced cancer-specific mortality.^24,25^ We assessed social network size by combining numerical responses to 2 questions: (1) “How many close friends do you have? That is, people that you feel at ease with, can talk to about private matters, and can call on for help?” and (2) “How many relatives do you have that you feel close to?” The residential segregation indices were created using the approach described by Massey and Denton.^26^ Geocoded residential addresses of participants at baseline were linked to 2010 US Census data to calculate 3 residential segregation measures (dissimilarity index, isolation index, and interaction index) at the census tract level. The dissimilarity index measures the evenness of racial distributions across a spatial unit (score range, 0-1, with higher scores indicating more racial residential segregation). The isolation index measures the extent to which Black adults are only exposed to one another (score range, 0-1, with higher scores indicating more racial residential segregation). The interaction index quantifies the extent to which Black adults interact with White adults (score range, 0-1, with higher scores indicating less racial residential segregation).

### Statistical Analysis

First, we compared differences in baseline characteristics of Black and White participants in the REGARDS study using χ^2^ tests for categorical variables and analysis of variance for continuous variables. We also assessed whether the racial residential segregation variables (dissimilarity, interaction, and isolation indices) were collinear using the variance inflation factor. Next, we examined unadjusted and adjusted differences between the proportion of Black vs White participants eligible for lung cancer screening according to 2013 and 2021 guidelines using modified Poisson models with robust SEs and an identity link. With a binary outcome (screening eligibility), the group means generated from these models represented the proportion of individuals in each group (Black vs White) who experienced the outcome. We first estimated unadjusted models, then added covariates to calculate adjusted estimates (eTable 1 and eTable 2 in the Supplement). We adjusted for covariates that were significantly different between Black and White participants.

All model results were presented as percentage point differences with 95% CIs. Analyses were conducted using SAS software, version 9.4 (SAS Institute Inc), and Stata software, version 14.2 (StataCorp LLC). Statistical tests were 2-sided, with a significance threshold of *P* = .05.

## Results

### Participant Characteristics

Of 14 285 REGARDS participants included in the final analytic sample, the mean (SD) age at baseline was 64.7 (7.5) years; 7675 participants (53.7%) were male, 6610 (46.3%) were female, 5787 (40.5%) were Black, 8498 (59.5%) were White, and 8946 (62.6%) were married (Table 1). Compared with participants who self-identified as White, those who self-identified as Black were significantly more likely to have low annual household income (<$20 000: 1598 participants [27.6%] vs 1081 participants [12.7%]; *P* < .001), have lower educational level (less than high school: 1205 participants [20.8%] vs 722 participants [8.5%]; *P* < .001), and reside in an urban region (4950 of 5385 participants [91.9%] vs 5217 of 7549 participants [69.1%]; *P* < .001). White participants generally had larger social networks compared with Black participants (eg, ≥15 close friends and relatives: 2553 of 8458 participants [30.2%] vs 1422 of 5742 participants [24.8%]; *P* < .001). Among the overall sample, 3765 participants (26.4%) reported being current smokers, and 10 520 participants (73.6%) reported being former smokers; however, a significant difference between Black vs White participants was observed regarding the proportion of current smokers (1817 participants [31.4%] vs 1948 participants [22.9%]; *P* < .001) and the number of pack-years among current smokers (median [IQR], 20.0 [8.8-40.0] pack-years vs 39.4 [21.0-53.0] pack-years; *P* < .001).

**Table 1.** Baseline Characteristics of Reasons for Geographic and Racial Differences in Stroke Study Participants

Characteristic	Participants, No./total No. (%)			*P* value
	Total (N = 14 285)	Black race (n = 5787)	White race (n = 8498)	
Age, mean (SD), y	64.7 (7.5)	64.0 (7.4)	65.2 (7.5)	<.001
Sex				
Female	6610/14 285 (46.3)	3065/5787 (53.0)	3545/8498 (41.7)	<.001
Male	7675/14 285 (53.7)	2722/5787 (47.0)	4953/8498 (58.3)	
Household income <$20 000	2679/14 285 (18.8)	1598/5787 (27.6)	1081/8498 (12.7)	<.001
Educational level less than high school	1927/14 285 (13.5)	1205/5787 (20.8)	722/8498 (8.5)	<.001
Married	8946/14 285 (62.6)	2881/5787 (49.8)	6065/8498 (71.4)	<.001
Has health insurance	13 304/14 285 (93.1)	5226/5787 (90.3)	8078/8498 (95.1)	<.001
Region of residence				
Stroke belt	4901/14 285 (34.3)	1853/5787 (32.0)	3048/8498 (35.9)	<.001
Stroke buckle	2902/14 285 (20.3)	893/5787 (15.4)	2009/8498 (23.6)	
Non–stroke belt	6482/14 285 (45.4)	3041/5787 (52.5)	3441/8498 (40.5)	
Rurality of residence				
Rural (≤25% urban)	1361/12 934 (10.5)	174/5385 (3.2)	1187/7549 (15.7)	<.001
Mixed (>25% to <75% urban)	1406/12 934 (10.9)	261/5385 (4.8)	1145/7549 (15.2)	
Urban (≥75% urban)	10 167/12 934 (78.6)	4950/5385 (91.9)	5217/7549 (69.1)	
No. of close friends and relatives				
Mean (SD)	13.3 (14.8)	12.7 (15.6)	13.65 (14.1)	<.001
Quartile				
0-5	3321/14 200 (23.4)	1574/5742 (27.4)	1747/8458 (20.7)	<.001
6-8	3093/14 200 (21.8)	1289/5742 (22.4)	1804/8458 (21.3)	
9-14	3811/14 200 (26.8)	1457/5742 (25.4)	2354/8458 (27.8)	
≥15	3975/14 200 (28.0)	1422/5742 (24.8)	2553/8458 (30.2)	
SF-12 mental component summary score, mean (SD)	53.91 (8.75)	53.22 (9.28)	54.37 (8.34)	<.001
Racial residential segregation index score at census tract level, mean (SD)				
Dissimilarity	0.50 (0.18)	0.45 (0.16)	0.53 (0.18)	<.001
Interaction	0.35 (0.30)	0.12 (0.13)	0.50 (0.28)	<.001
Isolation	0.57 (0.32)	0.81 (0.17)	0.41 (0.30)	<.001
Smoking status				
Current	3765/14 285 (26.4)	1817/5787 (31.4)	1948/8498 (22.9)	<.001
Former	10 520/14 285 (73.6)	3970/5787 (68.6)	6550/8498 (77.1)	
Quit >15 y ago	7336/10 520 (69.7)	2613/3970 (65.8)	4723/6550 (72.1)	<.001
No. of pack-years, median (IQR)				
All smokers (current and former)	19.0 (5.5-39.0)	13.5 (4.3-30.0)	23.0 (7.5-43.0)	<.001
Current smokers	30.0 (13.0-46.3)	20.0 (8.8-40.0)	39.4 (21.0-53.0)	<.001

Black participants resided in census tracts with higher levels of residential segregation compared with White participants, as indicated by higher scores on the isolation index (mean [SD], 0.81 [0.17] points vs 0.41 [0.30] points; *P* < .001) and lower scores on the interaction index (mean [SD], 0.12 [0.13] points vs 0.50 [0.28] points; *P* < .001) (Table 1). The dissimilarity index was the only measure that suggested Black participants resided in a census tract that was less segregated than that of White participants (mean [SD], 0.45 [0.16] points vs 0.53 [0.18] points; *P* < .001). Isolation and interaction indices were highly correlated (Pearson *r* = −0.97; *P* < .001), and variance inflation factors for these 2 indices were 18.6 and 18.9, respectively. Therefore, we only included the interaction and dissimilarity indices in the final model.

### LDCT Screening Eligibility

According to the 2013 guidelines, 3422 total participants (24.0%) were eligible for LDCT screening. Eligibility differed significantly between Black and White participants, with 1109 of 5787 Black participants (19.2%) meeting eligibility criteria compared with 2313 of 8498 White participants (27.2%). According to the 2021 guidelines, 4607 total participants (32.3%) would have been eligible for screening, with 1667 of 5787 Black participants (28.8%) meeting eligibility criteria vs 2940 of 8498 White participants (34.6%). In 2013, the unadjusted difference between Black and White participants was −8.06 percentage points (95% CI, −9.44 to −6.67 percentage points); in 2021, the difference decreased to −5.73 percentage points (95% CI, −7.28 to −4.19 percentage points) (Table 2).

**Table 2.** Differences in Eligibility for Lung Cancer Screening Between Black and White Participants in the Reasons for Geographic and Racial Differences in Stroke Study According to 2013 and 2021 US Preventive Services Task Force Guidelines

Model	Percentage point difference between Black vs White participants (95% CI)^a^	
	2013 Guidelines	2021 Guidelines
Unadjusted	−8.06 (−9.44 to −6.67)	−5.73 (−7.28 to −4.19)
Model 1: all variables in unadjusted model plus age at baseline and sex	−7.67 (−9.08 to −6.26)	−6.21 (−7.77 to −4.64)
Model 2: all variables in model 1 plus educational level, annual household income, and social network size	−9.92 (−11.36 to −8.47)	−9.08 (−10.67 to −7.48)
Model 3: all variables in model 2 plus marital status, insurance status, region of residence, and rurality of residence	−10.08 (−11.68 to −8.49)	−9.52 (−11.28 to −7.77)
Fully adjusted: all variables in model 3 plus residential segregation indices (interaction and dissimilarity)^b^^,^^c^	−12.66 (−14.71 to −10.61)	−12.15 (−14.37 to −9.93)

^a^ All differences were statistically significant at *P* < .001.

^b^ Fully adjusted model included age at baseline, sex, highest level of education, annual household income, social network size (number of friends and relatives), marital status, health insurance status, region of residence (stroke belt, stroke buckle, or non–stroke belt), rurality of residence, and racial residential segregation indices (interaction and dissimilarity).

^c^ Scores for residential segregation indices were calculated on the census tract level by aggregating census block–level data.

When age at baseline and sex were added to the model, the eligibility gap between 2013 guidelines (−7.67 percentage points; 95% CI, −9.08 to −6.26 percentage points) and 2021 guidelines (−6.21 percentage points; 95% CI, −7.77 to −4.64 percentage points) decreased. However, with each subsequent addition of grouped covariates (group 1: educational level, annual household income, and social network size; group 2: marital status, insurance status, region of residence, and rurality of residence; group 3: residential segregation indices) to the model, the adjusted difference between Black and White participants increased; in the final fully adjusted model, the eligibility gap was −12.66 percentage points (95% CI, −14.71 to −10.61 percentage points) in 2013 and −12.15 percentage points (95% CI, −14.37 to −9.93 percentage points) in 2021 (Table 2).

## Discussion

This cohort study found that although the 2021 revised USPSTF screening recommendations were designed to reduce disparities in screening eligibility, after application of the updated guidelines to diverse clinical populations, the lower age and pack-year thresholds improved but did not eliminate racial disparities. Hence, it remains important to consider other factors associated with the differences. To our knowledge, this study was the first to compare LDCT screening eligibility according to 2013 and 2021 USPSTF recommendations among non-Hispanic Black and White participants in the REGARDS cohort. The inclusion of the REGARDS cohort has notable implications because the study oversampled individuals from the southeastern stroke belt, a region with high state- and county-level residential segregation. This region also includes many states with smoking rates that are higher than the national average, some of which have the highest smoking rates in the world.^27^

Reasons for persistently disparate screening eligibility rates are multifactorial.^28,29^ For example, Black adults are more likely to be current smokers and to have lower quit rates, in part because of targeted marketing by the tobacco industry, particularly with regard to mentholated products.^30^ Although moderation or discontinuation of the smoking cessation criterion has been reported to increase eligibility among Black adults,^31^ eliminating the criterion alone has not entirely mitigated the screening disparity.^32^ An alternative model superior to pack-year standards is individual risk assessment using lung cancer risk prediction models.^28,33^ In one study conducted by Tammemägi et al,^34^ predictive models that were inclusive of risk factors such as educational attainment and high smoking intensity (average number of cigarettes smoked per day), had greater sensitivity and positive predictive values while preserving specificity for lung cancer detection compared with NLST criteria. However, few studies have developed predictive models solely among high-risk groups. Etzel et al^35^ used a multivariable risk model that was tested only among Black adult smokers with and without lung cancer, finding good to moderate discrimination (area under the curve, 79% [95% CI, 0.70%-0.88%] among those with lung cancer and 66% [95% CI, 0.61%-0.71%] among those without lung cancer). Thus, developing more ethnicity-specific lung cancer risk prediction models has the potential to improve screening among high-risk groups.

Black participants in our study were also significantly more likely to live in a racially segregated census tract than their White counterparts, which other studies have reported to be correlated with cancer stage, treatment, and survival. A survey by Annesi et al^16^ of data from Black and White patients with non–small cell lung cancer in the Surveillance, Epidemiology, and End Results database revealed that greater residential segregation among Black patients was associated with a higher likelihood of advanced-stage lung cancer diagnosis, a lower likelihood of surgical resection, and lower cancer-specific survival compared with White patients. Few studies have investigated the consequences of residential segregation for cancer screening. To our knowledge, the present study is the first to assess an association with lung cancer screening. Racial residential segregation is only 1 measure and consequence of racial discrimination. Although the mechanism and broad reach of structural and systemic racism as a fundamental factor in health inequities is beyond the scope of this paper and has been discussed elsewhere,^36,37^ the potential association cannot be overlooked. The modifiable policies and practices that collectively constitute and mutually uphold structural and systemic racism have implications for variations in disease risk (eg, smoking patterns and behaviors), opportunities and resource allocation, and access to health care services such as screening.^38^

Our additional finding that Black participants were significantly more likely to be younger than White participants has implications for screening access. Although Medicare universally covers LDCT lung screening, the same is not true for Medicaid and commercial health care plans across all states. Lozier et al^39^ found that among 13 380 adults who self-identified as non-Hispanic Black or non-Hispanic White and were eligible for screening based on the 2021 USPSTF guidelines, only an estimated 15% completed screening. Almost 80% of Black adults who completed screening were aged 65 to 80 years (Medicare age),^39^ suggesting insurance status may be a substantial barrier to screening. Therefore, expanding lung screening eligibility to include more minoritized groups and more groups with low socioeconomic status may perpetuate or perhaps even worsen disparities in the absence of health care reform.

Raising awareness and increasing knowledge about the benefits of lung cancer screening are also important elements in closing the disparity gap. Lack of awareness of LDCT screening recommendations is still pervasive among physicians and patients and may be factors in the low levels of LDCT screening uptake.^40,41,42^ These points highlight the need for multilevel efforts to eliminate racial screening disparities.

### Strengths and Limitations

This study has several strengths. The study used data from a large national cohort with substantial representation among Black adults, conducted rigorous data collection, and included structural factors associated with health such as residential segregation.

This study also has limitations. First, the measures of residential segregation at the census tract level do not capture the full context of individuals’ daily activities in spaces outside their residential neighborhoods (ie, workplaces) and social interactions that have direct consequences for important factors, such as composition of the social network, educational attainment, and access to health care.^43^ The indices also do not account for the duration of exposure (ie, how long a participant has lived in that census tract) across the life span or the arbitrary changes made to the boundaries of census tracts in response to population growth.^44^ Second, the REGARDS inclusion criteria requiring that participants have a home and telephone number precluded the most vulnerable members of the population from participating in the study. Although the REGARDS cohort is a large sample, it is not nationally representative; thus, the observed rates are specific to the population included. Third, our finding that the racial screening gap increased after adjusting our models for social factors associated with health suggests an independent association with important screening criteria such as the number of pack-years of smoking. Because Black participants experience disproportionate consequences from factors such as residential segregation, the racial gap becomes accentuated.

## Conclusions

The findings of this cohort study suggest that although expansion of the USPSTF lung cancer screening eligibility criteria was an important step to address racial differences in screening, without broader political and socioeconomic policy changes that address structural and systemic racism, the intended results of these changes may not be achieved.

## Notes

Supplement. **eTable 1.** Differences in Eligibility for Lung Cancer Screening Between Black and White Participants in the REGARDS Study According to USPSTF 2013 Guidelines **eTable 2.** Differences in Eligibility for Lung Cancer Screening Between Black and White Participants in the REGARDS Study According to USPSTF 2021 Guidelines
