# Supplementary material for: Analysis of Eligibility for Lung Cancer Screening by Race After 2021 Changes to US Preventive Services Task Force Screening Guidelines
Source: JAMA Netw Open. 2022 Sep 2;5(9):e2229741. doi: 10.1001/jamanetworkopen.2022.29741 (PMC9440399; doi:10.1001/jamanetworkopen.2022.29741)
Supplement: Supplement. — eTable 1. Differences in Eligibility for Lung Cancer Screening Between Black and White Participants in the REGARDS Study According to USPSTF 2013 Guidelines eTable 2. Differences in Eligibility for Lung Cancer Screening Between Black and White Participants in the REGARDS Study According to USPSTF 2021 Guidelines [file jamanetwopen-e2229741-s001.pdf]

Supplementary Online Content

Pinheiro LC, Groner L, Soroka O, et al. Analysis of eligibility for lung cancer screening by race after 2021 changes to US Preventive Services Task Force screening guidelines. *JAMA Netw Open*. 2022;5(9):e2229741. doi:10.1001/jamanetworkopen.2022.29741

eTable 1. Differences in Eligibility for Lung Cancer Screening Between Black and White Participants in the REGARDS Study According to USPSTF 2013 Guidelines

eTable 2. Differences in Eligibility for Lung Cancer Screening Between Black and White Participants in the REGARDS Study According to USPSTF 2021 Guidelines

This supplementary material has been provided by the authors to give readers additional information about their work.

© 2022 Pinheiro LC et al. *JAMA Network Open*.

eTable 1. Differences in Eligibility for Lung Cancer Screening Between Black and White Participants in the REGARDS Study According to USPSTF 2013 Guidelines

models					
	**Crude**	**1**	**2**	**3**	**Fully adjusted**
Race (ref=White)					
Black	-0.081 (-0.094, -0.067)	-0.077 (-0.091, -0.063)	-0.099 (-0.114, -0.085)	-0.101 (-0.117, -0.085)	-0.127 (-0.147, -0.106)
Age		0.0008 (-0.0001, 0.0016)	0.0004 (-0.0005, 0.0013)	0.0003 (-0.0006, 0.0012)	0.0004 (-0.0006, 0.0013)
Self-reported gender (ref= Male)					
Female		-0.027 (-0.041, -0.012)	-0.040 (-0.054, -0.026)	-0.055 (-0.071, -0.040)	-0.054 (-0.07, -0.038)
Education (ref=less than HS)					
High School or above			-0.065 (-0.088, -0.043)	-0.069 (-0.092, -0.045)	-0.067 (-0.091, -0.044)
Income (ref=less than $20k)					
More than or equal $20K			-0.084 (-0.104, -0.064)	-0.055 (-0.077, -0.034)	-0.054 (-0.076, -0.032)
Total # of close friends/relatives (ref=15 or more)					
0-5			0.049 (0.030, 0.069)	0.047 (0.026, 0.067)	0.045 (0.024, 0.066)
6-8			0.040 (0.020, 0.060)	0.034 (0.013, 0.055)	0.033 (0.012, 0.054)
9-14			0.015 (-0.003, 0.034)	0.014 (-0.005, 0.034)	0.014 (-0.006, 0.033)
Insurance (ref= presence)					
No insurance				0.050 (0.033, 0.067)	0.047 (0.030, 0.064)
Marital status (ref = Married)					
Non-married				-0.003 (-0.034, 0.028)	-0.004 (-0.035, 0.027)
Region (ref=Non-belt)					
Belt				0.009 (-0.008, 0.026)	0.002 (-0.015, 0.019)
Buckle				-0.008 (-0.028, 0.013)	-0.020 (-0.040, 0.001)
Size of census tract where participant lives [ref=Urban (>=75% urban)]					
Mixed (25-75% urban)				0.009 (-0.017, 0.035)	0.017 (-0.011, 0.045)
Rural (<=25% urban)				-0.002 (-0.027, 0.023)	0.008 (-0.018, 0.033)
Interaction index					-0.07 (-0.104, -0.036)
Dissimilarity index					-0.015 (-0.061, 0.032)

© 2022 Pinheiro LC et al. *JAMA Network Open*.

eTable 2. Differences in Eligibility for Lung Cancer Screening Between Black and White Participants in the REGARDS Study According to USPSTF 2021 Guidelines

	models				
	**Crude**	**1**	**2**	**3**	**Fully adjusted**
Race (ref=White)					
Black	-0.057 (-0.073, -0.042)	-0.062 (-0.078, -0.046)	-0.091 (-0.107, -0.075)	-0.095 (-0.113, -0.078)	-0.122 (-0.144, -0.099)
Age		-0.0049 (-0.0059, -0.0039)	-0.0053 (-0.0063, -0.0043)	-0.0055 (-0.0066, -0.0044)	-0.0054 (-0.0065, -0.0043)
Self-reported gender (ref= Male)					
Female		-0.007 (-0.023, 0.009)	-0.025 (-0.041, -0.009)	-0.049 (-0.067, -0.032)	-0.048 (-0.065, -0.03)
Education (ref=less than HS)					
High School or above			-0.078 (-0.102, -0.054)	-0.084 (-0.109, -0.058)	-0.083 (-0.109, -0.057)
Income (ref=less than $20k)					
More than or equal $20K			-0.107 (-0.128, -0.085)	-0.076 (-0.099, -0.052)	-0.075 (-0.098, -0.051)
Total # of close friends/relatives (ref=15 or more)					
0-5			0.074 (0.052, 0.095)	0.069 (0.046, 0.092)	0.067 (0.045, 0.09)
6-8			0.052 (0.03, 0.074)	0.045 (0.023, 0.068)	0.045 (0.022, 0.068)
9-14			0.023 (0.003, 0.043)	0.022 (0.001, 0.043)	0.021 (0, 0.042)
Insurance (ref= presence)					
No insurance				0.072 (0.053, 0.091)	0.069 (0.049, 0.088)
Marital status (ref = Married)					
Non-married				-0.015 (-0.049, 0.02)	-0.016 (-0.05, 0.019)
Region (ref=Non-belt)					
Belt				0.008 (-0.011, 0.026)	0.0002 (-0.019, 0.019)
Buckle				-0.003 (-0.026, 0.019)	-0.016 (-0.039, 0.007)
Size of census tract where participant lives [ref=Urban (>=75% urban)]					
Mixed (25-75% urban)				0.006 (-0.022, 0.034)	0.016 (-0.014, 0.046)
Rural (<=25% urban)				-0.015 (-0.042, 0.012)	-0.004 (-0.032, 0.024)
Interaction index					-0.071 (-0.108, -0.035)
Dissimilarity index					-0.024 (-0.075, 0.027)

Data presented as rates difference between Black and White participants and 95% Confidence Intervals

Model 1: Crude + age at baseline, gender

Model 2: Model 1 + education, annual income, social network

Model 3: Model 2+ marital status, insurance, region, rural residence

Fully adjusted: model 3+ residential segregation indices (interaction, dissimilarity)

© 2022 Pinheiro LC et al. *JAMA Network Open*.

Abbreviations: REGARDS, Reasons for Geographic and Racial Differences in Stroke; USPTF, U.S. Preventive Task Force
Fully adjusted model includes covariates: age at baseline, gender, highest level of education attained, annual household income, social network size (# of friends, relatives), marital status, health insurance status, region (stroke belt, stroke buckle, non-belt), rurality, and racial residential segregation indices (interaction, dissimilarity).
All covariates were calculated at baseline.
Residential segregation indices were calculated on census tract level by aggregating census block level data

© 2022 Pinheiro LC et al. *JAMA Network Open*.
